# High‐Phenolic Cocoa Enhances Drug‐Induced Reinstatement of Cocaine‐Conditioned Place Preference Independently of Increasing Adult Hippocampal Neurogenesis

**DOI:** 10.1002/fsn3.70842

**Published:** 2025-09-14

**Authors:** Sonia Melgar‐Locatelli, María del Carmen Mañas‐Padilla, Patricia Rivera, Celia Rodríguez‐Pérez, Estela Castilla‐Ortega

**Affiliations:** ^1^ Departamento de Psicobiología y Metodología de las Ciencias del Comportamiento Universidad de Málaga Málaga Spain; ^2^ Instituto de Investigación Biomédica de Málaga y Plataforma en Nanomedicina‐IBIMA Plataforma BIONAND Málaga Spain; ^3^ Departamento de Nutrición y Bromatología Universidad de Granada, Campus Universitario de Cartuja Granada Spain; ^4^ Instituto de Nutrición y Tecnología de los Alimentos ‘José Mataix’ (INYTA), Centro de Investigación Biomédica Universidad de Granada Granada Spain; ^5^ Universidad Internacional de la Rioja (UNIR) Rioja Spain; ^6^ Unidad de Gestión Clínica de Salud Mental Hospital Regional Universitario de Málaga Málaga Spain; ^7^ Departamento de Anatomía Humana, Medicina Legal e Historia de la Ciencia Universidad de Málaga Málaga Spain; ^8^ Instituto de Investigación Biosanitaria de Granada (ibs.GRANADA) Granada Spain

**Keywords:** addiction, anxiety, dopamine, hippocampus, neuroplasticity, reinstatement

## Abstract

Cocaine is a powerful psychostimulant that disrupts brain function, affecting both physical and mental health. Natural cocoa, rich in polyphenols, influences neuroplasticity and cognitive processes. This study examined the effects of a high‐phenolic cocoa‐enriched diet on mice previously exposed to cocaine in a conditioned place preference (CPP) paradigm, assessing CPP maintenance, cognitive and emotional behavior, and adult hippocampal neurogenesis (AHN) after protracted abstinence. Forty‐two male and female C57BL/6JRj mice were divided into saline (VEH) and cocaine (COC) groups. The CPP paradigm included pre‐conditioning, 2 weeks of conditioning with alternating cocaine (20 mg/kg) and saline pairings, and a post‐conditioning test. COC mice then received either a 10% cocoa‐enriched diet or regular diet. After 24 days, mice underwent a second CPP test session and a cocaine‐induced reinstatement session (10 mg/kg). Behavioral assessments and immunohistochemistry for AHN‐related markers followed. While cocaine did not produce long‐term emotional and cognitive changes, it reduced the survival of adult‐born cells differentiating into mature neurons. Cocoa consumption did not influence the long‐term CPP maintenance but significantly increased cocaine‐induced reinstatement and heightened subsequent anxiety‐like behavior in the COC mice. Additionally, cocoa‐fed mice showed enhanced AHN; however, mediation analysis confirmed that neurogenesis did not influence drug‐seeking. No sex differences were observed. These findings suggest that a cocoa‐rich diet may modulate addiction‐related behavior through pathways independent of AHN. Although cognitive, emotional, and neuroprotective benefits are associated with cocoa consumption, its role in cocaine addiction requires further investigation, as there is a potential risk of cocoa interacting with drug reward or drug‐seeking.

## Graphical abstract

High‐phenolic cocoa consumption enhanced cocaine‐induced reinstatement in a conditioned place preference paradigm. While cocoa promoted adult hippocampal neurogenesis, this effect did not mediate drug‐seeking behavior, suggesting a complex interaction between cocoa and cocaine on the reward system

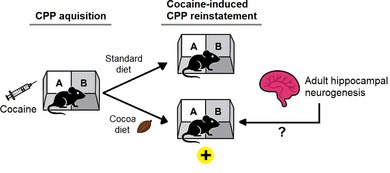

## 1 Introduction

Cocaine addiction is a rising global public health concern, characterized by enduring behavioral dysfunctions that compromise physical health, interpersonal relationships, and economic stability (Dang et al. 2022). As addiction develops, the motivation for cocaine use shifts from seeking its pleasurable effects to engaging in compulsive, habitual behaviors. These behaviors are strongly influenced by environmental cues repeatedly associated with the drug's rewarding properties. Contextual cues can provoke intense cravings, driving compulsive drug‐seeking behavior, increasing relapse risk, and contributing to the chronic nature of the disorder (Ndiaye et al. 2024). Consequently, cocaine addiction is increasingly recognized as a disorder of impaired cognition, wherein persistent drug‐associated memories foster maladaptive behaviors. The enduring impact of memories tied to cocaine‐associated stimuli can be partly attributed to their resistance to extinction and forgetting (Kutlu and Gould 2016). Furthermore, persistent cocaine exposure often involves a cognitive impairment affecting the ability to form new, adaptive memories, thereby reinforcing drug‐seeking patterns (Castilla‐Ortega et al. 2017; Regnier et al. 2022).

The hippocampus plays a central role in the formation and persistence of these drug‐related memories. As a key brain region involved in both declarative and associative memory processes, it is intricately linked to neural circuits underlying cocaine addiction (Castilla‐Ortega, Serrano, et al. 2016). Clinical studies show that individuals with cocaine addiction exhibit heightened hippocampal activity in response to drug‐associated cues, and this hyperactivity correlates with the intensity of cravings (Castilla‐Ortega, Serrano, et al. 2016). Preclinical research investigating the association between drugs and environmental contexts frequently employs the conditioned place preference (CPP) paradigm. This approach has consistently shown that the hippocampus is critically involved in the acquisition, extinction, retention, and reinstatement of contextual cocaine‐associated memories (Bender and Torregrossa 2020; Burgdorf et al. 2017; Hitchcock and Lattal 2018). Within the hippocampus, adult hippocampal neurogenesis (AHN) refers to the continuous generation of new neurons in the dentate gyrus throughout adulthood. These newly formed neurons are thought to play a crucial role in cognitive flexibility, pattern separation, and adaptive learning (Fölsz et al. 2023). Notably, AHN has been implicated in both memory formation and forgetting processes, regulating interference between overlapping memories (Chang and Hen 2024; Scott et al. 2021). In addition, emerging evidence suggests that AHN contributes to the suppression of maladaptive drug‐associated memories. Suppressing AHN has been shown to increase cocaine‐seeking and self‐administration behavior (Castilla‐Ortega, Blanco, et al. 2016; Deroche‐Gamonet et al. 2019; Luján et al. 2020), whereas enhancing AHN—particularly through pharmacological or genetic stimulation—can facilitate the attenuation of previously acquired cocaine‐associated memories (Ladrón de Guevara‐Miranda et al. 2019; Li et al. 2021).

Recent findings suggest that dietary interventions may influence neuroplasticity and cognitive function. Cocoa, a compound widely recognized for its health benefits (Cory et al. 2018), has been shown to improve memory and promote AHN in healthy male and female mice, potentially through the action of its flavanol constituents (Melgar‐Locatelli, Mañas‐Padilla, Castro‐Zavala, et al. 2024). However, the role of cocoa‐induced AHN in modulating drug‐context associations remains unclear. The present study aims to investigate the effects of a polyphenol‐rich cocoa diet on the persistence of drug‐context associations in a cocaine‐conditioned environment, specifically assessing CPP maintenance, cocaine‐induced relapse, and the potential mediating role of AHN. Additionally, we evaluated cognitive and emotional function through a comprehensive behavioral test battery.

## 2 Material and Methods

### 2.1 Animals

Forty‐two male and female C57BL/6JRj mice (7 weeks old) were acclimated for 1 week before the experiment. They were individually housed under standard conditions (22°C ± 2°C; 12‐h light/dark cycle; lights on at 8:00 a.m.) with nesting material and ad libitum access to food and water. To confirm estrous cyclicity, a 5‐day vaginal cytology protocol was performed in 50% of female mice, starting 5 days prior to sacrifice. Food intake and body weight were monitored weekly during the first 4 weeks following diet administration.

Procedures followed European (Directive 2010/63/UE) and Spanish (Real Decreto 53/20130, Ley 32/2007) regulations for animal research and were approved by the University of Málaga ethics committee (code: 104‐2021‐A) and Junta de Andalucía (code: 3/11/2021/170).

### 2.2 Conditioned Place Preference and Diet Administration

Mice were initially assigned to vehicle (VEH, *n* = 14; 7 males and 7 females) or cocaine (COC, *n* = 28; 14 males and 14 females) groups, balanced by sex. A prolonged CPP published protocol (Mañas‐Padilla et al. 2021) was used to assess cocaine‐associated memory and induce cognitive deficits.

**FIGURE 1:**
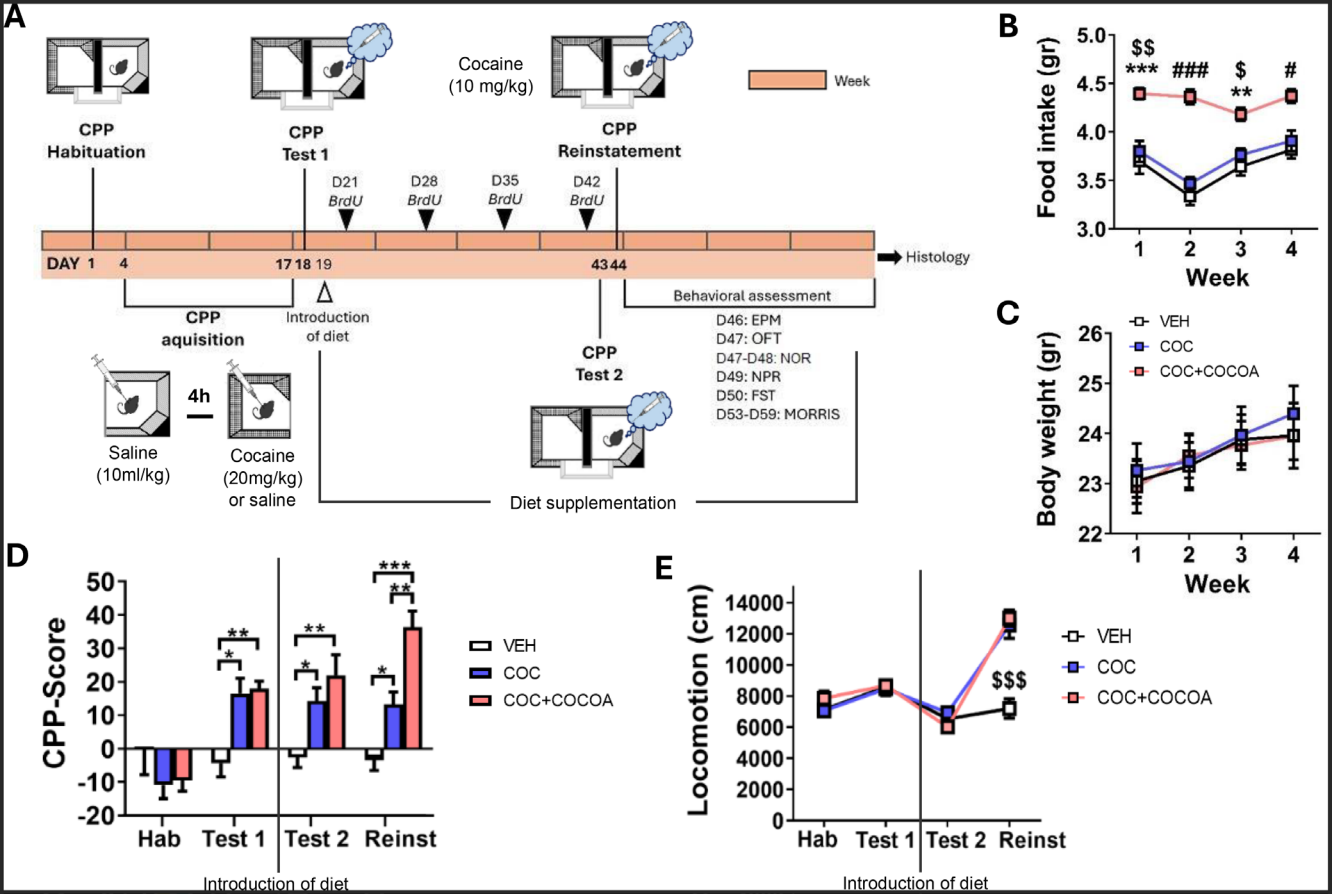
(A) Experiment schedule. Mice underwent a 14‐day chronic conditioned place preference (CPP) task with cocaine (cocaine mice) or saline (VEH mice). After confirming cocaine‐induced CPP acquisition (Test 1), cocaine mice were then assigned to either a standard diet (COC) or a cocoa‐enriched (COC + COCOA) treatment. All groups weretested for long‐term cocaine‐CPP memory (Test 2), cocaine‐induced reinstatement (Reinst), and emotional/cognitive behavior. (B) Mice receiving the cocoa‐enriched diet showed increased food intake over the 4‐week period compared to those on a standard diet. (C) Despite the increased intake, cocoa consumption did not significantly affect body weight gain. (B, C) Post hoc least significant difference (LSD): Difference between the COC + COCOA group versus the VEH group: *p ≤ 0.05; **p ≤ 0.005; ***p ≤ 0.001; difference between the COC + COCOA group versus the COC group: ^$^ *p* ≤ 0.05; ^$$^ *p* ≤ 0.005; $$$*p* ≤ 0.001; difference between the COC + COCOA group versus the other two groups: ^#^ *p* ≤ 0.05; ^###^ *p* ≤ 0.001. Data are expressed as mean ± SEM. (D) While CPP acquisition was similar across cocaine groups, the cocoa‐fed group showed increased preference for the cocaine‐paired compartment during reinstatement. (E) Both cocaine groups exhibited increased locomotor activity during reinstatement. Hab, habituation session; DCX, doublecortin; EPM, elevated plus maze; FST, forced swimming test; NOR, novel object recognition; NPR, novel place recognition; OFT, open field test; PCNA, proliferating cell nuclear antigen.

On Day 1 (Figure 1A), 10‐week‐old mice underwent CPP habituation, receiving an intraperitoneal (i.p.) saline injection (0.9% NaCl, 10 mL/kg) and exploring the apparatus freely for 20 min. During CPP acquisition (Days 4–17), COC mice underwent two daily conditioning sessions (cocaine‐ and saline‐paired) with alternating order (“morning” or “afternoon”) and a 4‐h interval (Mañas‐Padilla et al. 2021). In the cocaine‐paired session, COC mice received 20 mg/kg cocaine (i.p., Alcaliber S.A., Madrid, Spain; saline‐diluted at 10 mL/kg) in the restricted cocaine‐paired compartment for 15 min. In the saline‐paired session, they received an equivalent saline volume in the opposite compartment for the same duration. VEH mice received saline in both sessions. Twenty‐four hours after the final conditioning session, a post‐conditioning test (Test 1), was conducted, identical to habituation. COC mice of both sexes were then randomly assigned to either a standard diet (COC group) or a cocoa‐enriched diet (COC + COCOA group) until the experiment's end. The selected cocoa powder had the highest polyphenol content among previously tested commercial samples (Melgar‐Locatelli, Mañas‐Padilla, Castro‐Zavala, et al. 2024; Razola‐Díaz et al. 2023). The cocoa‐supplemented diet, formulated by Scientific Animal Food and Engineering (SAFE, Bourgogne, France), contained 10% high‐phenolic cocoa powder in standard pellets (SAFE D40). After 24 days of dietary intervention, a second post‐conditioning test (Test 2, Day 43) assessed CPP retention and cocoa's effect on memory persistence. On Day 44, a cocaine‐induced reinstatement session was performed, following the same procedure as the previous tests, using a priming dose of cocaine (10 mg/kg, i.p.). CPP score was calculated as: ([seconds spent in the cocaine‐paired compartment − seconds spent in the saline‐paired compartment]/seconds spent in both compartments) × 100%. A positive CPP score indicated preference for the cocaine‐associated compartment.

### 2.3 Behavioral and Cognitive Assessment

Behavioral, emotional, and cognitive assessments began 2 days after the CPP reinstatement test, following established protocols (Melgar‐Locatelli, Mañas‐Padilla, Castro‐Zavala, et al. 2024; Melgar‐Locatelli, Mañas‐Padilla, Gavito, et al. 2024). The elevated plus maze (EPM, Day 46) and open field test (OFT, Day 47) assessed anxiety‐like behavior and locomotion. Cognitive function was evaluated using the novel object recognition (NOR, Days 47–48) and novel place recognition (NPR, Day 49) tests. The forced swimming test (FST, Day 50) assessed despair‐like behavior. The water maze (Days 53–59) included habituation (Day 53), visible platform training (Days 53–54), spatial reference memory training with a hidden platform (Days 55–58) and a probe trial to evaluate short‐term retention 24 h after the last spatial training session (Day 59).

### 2.4 Bromodeoxyuridine Administration

To evaluate AHN, bromodeoxyuridine (BrdU, Sigma‐Aldrich, Madrid, Spain) was administered during the first 4 weeks of dietary intervention. Mice received two intraperitoneal injections of BrdU (75 mg/kg, saline‐diluted) per day, spaced 4 h apart on days 21, 28, 35, and 42 (Figure 1A), to label newly generated cells (Mañas‐Padilla et al. 2023).

### 2.5 Assessment of AHN

Three days after behavioral tests, mice were sacrificed via intracardiac perfusion (Melgar‐Locatelli, Mañas‐Padilla, Castro‐Zavala, et al. 2024). Free‐floating immunohistochemistry was performed on 45 μm coronal vibratome sections from the right hemisphere. Doublecortin (DCX) and proliferating cell nuclear antigen (PCNA) were measured using DAB staining, while BrdU/mature neuron co‐labeling was assessed through immunofluorescence, following the same antibodies and procedures cited in Melgar‐Locatelli, Mañas‐Padilla, Castro‐Zavala, et al. (2024). Cell quantification was conducted in the dentate gyrus of the dorsal hippocampus (bregma −1.06 to −3.08 mm).

### 2.6 Statistical Analysis

Initial analyses included sex as a factor, alongside treatment, but since no significant sex differences emerged, subsequent analyses were performed at the group level. One‐way analysis of variance (ANOVA) or repeated measures ANOVAs were performed, followed by post hoc Fisher's least significant difference (LSD) test, with statistical significance set at *p* ≤ 0.05. Non‐significant results are summarized in Table S1. Mediation analyses were performed using IBM SPSS 28 (IBM Corporation, Armonk, NY, USA) and the PROCESS macro (Hayes 2013) as described in Ladrón de Guevara‐Miranda et al. (2019). For both the COC and COC + COCOA treatment groups, a simple mediation model analyzed diet as a predictor, CPP retention/reinstatement (i.e., CPP‐Score in the retention session) as the outcome, and AHN [i.e., BrdU+/NeuN+ cells per mm^2^] as the mediator. All variables were standardized (mean = 0, SD = 1), and the analysis followed the causal steps approach (Baron and Kenny 1986). Bootstrapping (10,000 samples) with bias‐corrected 95% confidence intervals excluding zero was used to assess significance (*p* ≤ 0.05).

## 3 Results

### 3.1 Cocoa Supplementation Led to Increased Free Food Consumption

Assessment of food consumption during 4 weeks after the introduction of the experimental diet, revealed that mice fed a cocoa‐enriched diet exhibited a significantly higher average food intake (4.33 ± 0.07 g/day) compared to both the control (3.62 ± 0.10 g/day) and the COC groups (3.73 ± 0.09 g/day), which were maintained on a standard diet [repeated measures ANOVA “treatment × week”: effect for “treatment”: *F*(2, 39) = 33.162, *p* < 0.001; “week”: *F*(3, 117) = 10.421, *p* < 0.001; “treatment × week”: *F*(6, 117) = 3.088, *p* = 0.008; LSD post hoc analysis is shown in Figure 1B]. Despite this increase in food consumption, cocoa supplementation did not lead to significant changes in body weight gain (Figure 1C). During the analysis of sex differences, food consumption was not influenced by the sex of the animals (Figure S1). Nevertheless, male mice exhibited a higher body weight compared to females (Figure S2). These findings are consistent with those reported in our previous study (Melgar‐Locatelli, Mañas‐Padilla, Castro‐Zavala, et al. 2024).

### 3.2 Cocoa‐Enriched Diet Enhanced Cocaine‐Induced CPP Reinstatement

Chronic cocaine exposure led to a significant preference for the cocaine‐paired compartment [repeated‐measures ANOVA for CPP‐Score across the “habituation,” “test 1,” “test 2” and “reinstatement” sessions: “treatment”: *F*(2, 39) = 6.493, *p* = 0.004; “session,” *F*(3, 117) = 16.276, *p* < 0.001; “treatment × session,” *F*(6, 117) = 6.120, *p* < 0.001]. LSD post hoc analyses revealed no effect of cocoa consumption on long‐term CPP maintenance, as COC and COC + COCOA groups behaved similarly in Test 2. However, cocoa‐fed mice spent more time in the cocaine‐paired compartment during reinstatement (Figure 1D). Locomotion did not differ between cocaine‐treated groups across the CPP task (Figure 1E).

### 3.3 Cocoa‐Enriched Diet Increased AHN

**FIGURE 2:**
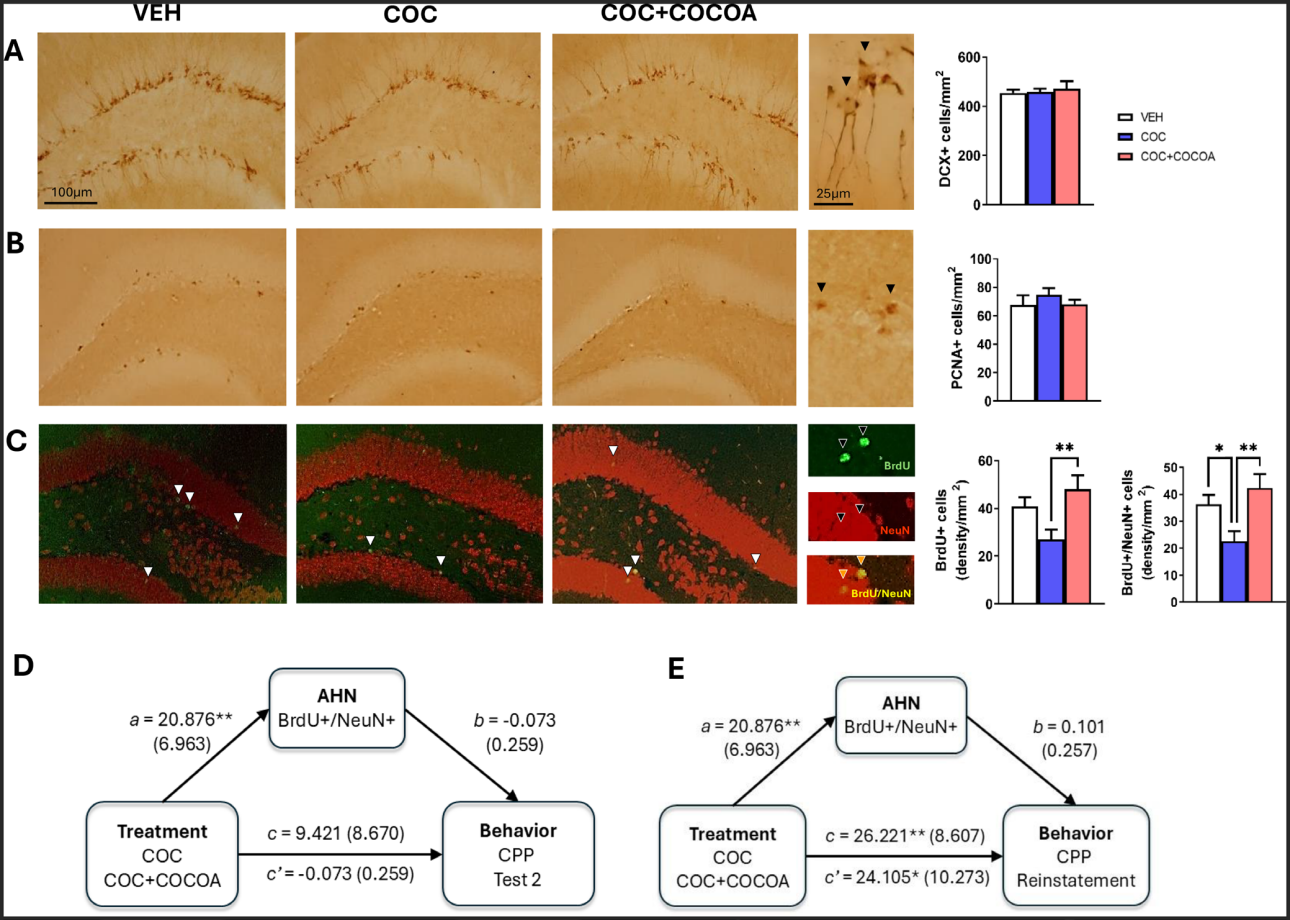
(A, B) No differences were observed in the number of immature neurons (DCX+) or proliferating cells (PCNA+). Black arrows indicate positive cells. (C) Cocoa‐fed mice showed enhanced adult hippocampal neurogenesis (AHN), with more BrdU+ cells differentiated into mature neurons (BrdU+/NeuN+). White arrows highlight BrdU+ nuclei, co‐labeled with NeuN. Black arrows indicate BrdU, NeuN, or both (highlighted in orange). (A–C) Post hoc least significant difference (LSD): *p ≤ 0.05, **p ≤ 0.01 (between‐group differences). Data are expressed as mean ± SEM. (D) Cocoa supplementation increased AHN during CPP test 2 (path a), but had no total (path c) or direct (path c′) effects on CPP retention, nor was AHN correlated with retention (path b). Bootstrapping confirmed no significant effect [*a* × *b* = −1.522, 95% CI (−13.297 to 9.325)]. (E) However, cocoa intake significantly influenced cocaine‐induced reinstatement both directly and through its total effect (paths c′ and c), despite no link between AHN and reinstatement (path b). Bootstrapping confirmed no mediation [*a* × *b* = 2.116, 95% CI (−6.306 to 11.094)]. Data are displayed as coefficients (standard error) with categorical values for treatments: 0 (COC) and 1 (COC + COCOA). Significant correlations: *p ≤ 0.05, **p ≤ 0.01.

Cocoa supplementation significantly increased adult hippocampal neurogenesis in cocaine‐abstinent mice. While no significant differences were observed in DCX or PCNA expression (Figure 2A,B), analysis of BrdU and BrdU/NeuN biomarkers in 29 animals (after missing three hippocampal samples during BrdU immunostaining; two COC and one VEH) revealed that cocaine exposure significantly reduced the survival of newly born cells (BrdU+). Importantly, cocoa‐enriched diet alleviated this deficit, significantly increasing both BrdU+ cell survival [one‐way ANOVA: *F*(2, 36) = 4.947, *p* = 0.013] and the number of newly‐born hippocampal neurons, as evidenced by BrdU/NeuN double staining [one‐way ANOVA: *F*(2, 36) = 5.392, *p* = 0.009]. LSD post hoc analyses are represented in Figure 2C.

### 3.4 AHN Did Not Mediate Cocaine‐Induced Reinstatement

Causal mediation analyses examined whether AHN (BrdU+/NeuN+ cells) mediated long‐term CPP maintenance and reinstatement. Cocoa supplementation significantly increased AHN during CPP Test 2 (path a), but no total (path c) or direct (path c′) effects on CPP retention were observed, nor were AHN and CPP retention correlated (path b; Figure 2D). However, cocoa intake did have a significant impact on cocaine‐induced reinstatement, though this effect was independent of AHN (Figure 2E). These findings suggest that cocoa influenced cocaine‐induced reinstatement through alternative mechanisms unrelated to neurogenesis.

### 3.5 Cocoa‐Enriched Diet Increased Anxiety in Cocaine‐Exposed Mice With Minimal Effects on Water Maze Performance

**FIGURE 3:**
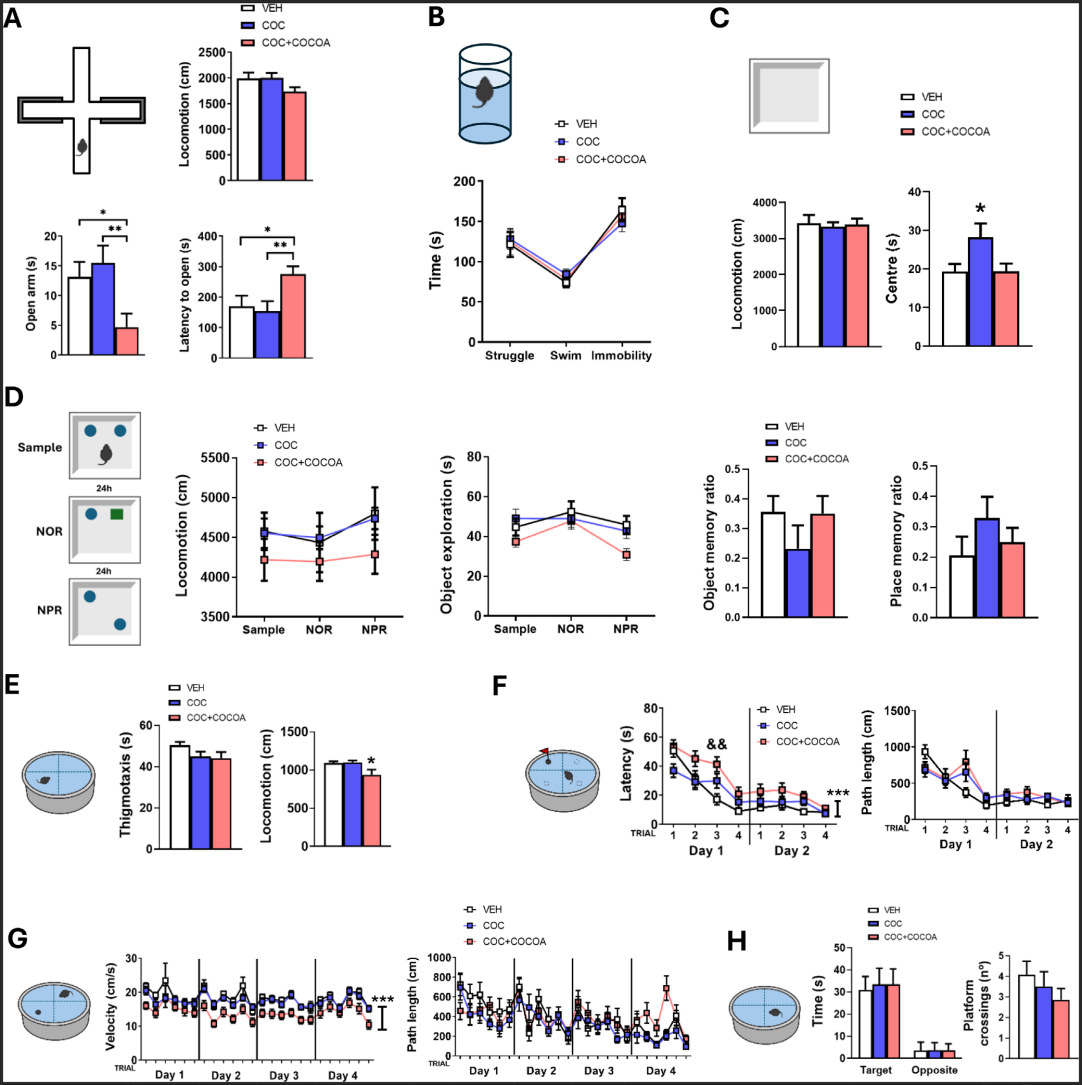
(A) The cocoa‐enriched diet increased anxiety‐like behavior in cocaine‐exposed mice in the EPM. (B) No effects were observed in the FST. (C) All groups displayed similar locomotion in the OFT, but COC mice spent more time in the center. (D) Locomotion, total exploration time, and memory performance in the NOR and NPR tests remained consistent across groups. (E) During water maze habituation, all groups exhibited similar exploratory behavior, though COC + COCOA mice showed reduced locomotor activity. (F, G) COC + COCOA mice demonstrated longer latencies to reach the platform and reduced swimming velocity during the visible platform and training tasks, while overall path length remained unaffected. (H) No significant differences were detected during the probe trial. Post hoc least significant difference (LSD): ^&&^ *p* ≤ 0.01 (VEH vs. COC + COCOA); *p ≤ 0.05, **p ≤ 0.01 (between‐group differences). Data are expressed as mean ± SEM.

Cocaine‐exposed mice on a cocoa‐enriched diet showed heightened anxiety‐like behavior in the EPM, spending less time in open arms [one‐way ANOVA: *F*(2, 39) = 4.737, *p* = 0.014; LSD post hoc analysis in Figure 3A] and exhibiting longer entry latencies [one‐way ANOVA: *F*(2, 39) = 4.495, *p* = 0.018; LSD post hoc analysis in Figure 3A], with no locomotor differences (Figure 3A). Neither cocaine nor cocoa diet affected FST behavior (Figure 3B). During OFT habituation, all groups exhibited similar locomotion, but COC mice spent more time in the center [one‐way ANOVA: *F*(2, 39) = 3.762, *p* = 0.032; LSD post hoc analysis in Figure 3C]. NOR and NPR tests showed no group differences in locomotion, exploration, or memory (Figure 3D). During water maze habituation, all groups spent similar time in periphery (Figure 3E), though COC + COCOA mice exhibited reduced locomotion [one‐way ANOVA: *F*(2, 39) = 4.460, *p* = 0.018; LSD post hoc analysis in Figure 3E]. These mice also demonstrated longer latencies to reach the platform [repeated‐measures ANOVA “treatment × trial”: “treatment”: *F*(2, 39) = 8.414, *p* = 0.001; “trial”: *F*(7, 273) = 38.729, *p* < 0.001; “treatment × trial”: *F*(14, 273) = 1.730, *p* = 0.050; LSD post hoc analysis in Figure 3F] and lower swimming velocity [repeated‐measures ANOVA “treatment × trial”: “treatment”: *F*(2, 39) = 8.321, *p* = 0.001; “trial”: *F*(23, 897) = 8.555, *p* < 0.001; “treatment × trial”: *F*(46, 897) = 0.751, *p* = 0.888; LSD post hoc analyses in Figure 3G] during visible platform and training sessions. However, distance traveled (i.e., path length until reaching the platform) remained unchanged in both tasks (Figure 3F,G), suggesting no learning impairments. No significant differences were detected in the time spent in the target and opposite quadrants or in the number of platform crossings during the probe trial (Figure 3H).

### 3.6 Exploration of Sex Differences

No sex differences were found in CPP task, cognitive behavior or AHN (Supporting Information). However, male mice exhibited reduced locomotor activity (979.47 ± 44.79 cm; Mean ± SEM) compared to females (1107.91 ± 24.44 cm) during water maze habituation [one‐way ANOVA: *F*(1, 40) = 6.339, *p* = 0.016; LSD post hoc analysis in Figure S8]. In the EPM, control females spent less time in the open arms than males, but this pattern was reversed with cocaine: females showed increased open‐arm time, while males showed a reduction. In the cocoa‐treated group, both sexes displayed similarly low open‐arm times [two‐way ANOVA: “treatment”: *F*(2, 36) = 5.846, *p* < 0.001; “sex”: *F*(1, 36) = 1.239, *p* = 0.273; “treatment × sex”: *F*(2, 36) = 5.448, *p* = 0.009; Figure S6B].

## 4 Discussion

This study explored the effects of a high‐phenolic cocoa‐enriched diet on addiction‐related behaviors, AHN, and cognitive and emotional responses in mice with previous chronic cocaine exposure. While cocaine may reduce cell proliferation in the dentate gyrus, this depletion may be limited to a short time after drug withdrawal (Castilla‐Ortega et al. 2017; Sudai et al. 2011). Consistent with this, PCNA and DCX expression in our study was similar between cocaine‐exposed and control mice, assessed ~7 weeks post‐cocaine administration. However, the survival of newly born hippocampal neurons (i.e., BrdU+ and BrdU/NeuN+ cell counts) was significantly reduced by cocaine, in line with previous studies in rats reporting impaired neurogenesis following repeated or chronic cocaine exposure (Andersen et al. 2007; Yamaguchi et al. 2004). While the effects of cocaine on cell proliferation appear to be transient, there is less agreement regarding its impact on neuronal survival, with mixed results likely reflecting differences in species, dosing regimens, and timing of assessment. For example, Mañas‐Padilla et al. (2021) found no significant long‐term reduction in the survival of newly born neurons in mice, highlighting the importance of protocol‐specific variables such as treatment duration and post‐cocaine delay.

A novel finding of this work is that AHN was effectively restored by a cocoa‐enriched diet in the cocaine‐abstinent animals, significantly increasing both BrdU+ cell survival and the number of newly born mature neurons. Accordingly, our prior research showed that supplementing the diet with the high‐phenolic cocoa powder used here promoted AHN in healthy male and female mice, an effect not observed with low‐phenolic cocoa, suggesting that cocoa polyphenols potentiate AHN (Melgar‐Locatelli, Mañas‐Padilla, Castro‐Zavala, et al. 2024). The functional implications of this AHN increase, however, appear complex. Increased AHN is expected to reduce CPP behavior by facilitating forgetting of previously acquired cocaine‐context associations and/or by reducing drug craving. For example, enhancing AHN with lysophosphatidic acid reduced long‐term cocaine CPP maintenance (Ladrón de Guevara‐Miranda et al. 2019). Similarly, physical and cognitive training, which stimulate AHN, accelerated CPP extinction and prevented reinstatement by low‐dose cocaine priming (Ávila‐Gámiz et al. 2025). Despite cocoa's enhancement of AHN, our results did not support its role in reducing CPP behavior. On the contrary, cocoa‐treated mice with increased AHN showed normal CPP maintenance and exacerbated CPP reinstatement when exposed to a cocaine prime. Mediation analyses confirmed no significant influence of BrdU+/NeuN+ cells on CPP retention or reinstatement, suggesting that cocoa's effects on CPP were likely driven by other mechanisms.

Importantly, previous studies used different AHN‐modulating strategies other than cocoa, probably triggering different mechanisms. For example, in addition to promoting AHN, we previously revealed that high‐phenolic cocoa increased hippocampal BDNF levels (Melgar‐Locatelli, Mañas‐Padilla, Castro‐Zavala, et al. 2024), which may modulate hippocampal participation in the reinstatement of CPP behavior. On a different note, since cocaine‐induced reinstatement is strongly linked to mesolimbic dopaminergic activation (Anderson and Pierce 2005), cocoa may have interacted with this system, potentiating cocaine reward and CPP behavior after a drug prime. Despite the absence of additional studies on the rewarding properties of natural cocoa in mice or rats, our data showed that mice receiving the cocoa‐enriched diet increased their intake, which may suggest a reinforcing property of natural cocoa itself. In line with this, cocoa‐derived products such as chocolate, or certain cocoa components such as caffeine—a major methylxanthine in cocoa—have been shown to exert rewarding effects, supporting CPP, self‐administration, and reinstatement behaviors in rodents (Muñiz et al. 2017; Noye Tuplin and Holahan 2019; Zaru et al. 2013). This is accompanied by neuroplastic changes in critical reward‐related regions, including the nucleus accumbens (Muñiz et al. 2017; Noye Tuplin and Holahan 2019). Further evidence supports a potential role of cocoa's compounds in modulating the effects of cocaine. Caffeine potentiates cocaine's reinforcing properties, as evidenced by increased CPP behavior when co‐administered intraperitoneally in mice (Muñiz et al. 2017) and rats (Bedingfield et al. 1998). Resveratrol—a polyphenol found in red wine but also in cocoa (Chkhikvishvili et al. 2022)—enhances cocaine‐stimulated dopamine neurotransmission in vitro (Shuto et al. 2013), and oral administration of either cocoa, theobromine, or caffeine increased cocaine‐induced hyperlocomotion (Kuribara and Tadokoro 1992).

Cocoa‐fed, cocaine‐abstinent mice also exhibited increased anxiety‐like behavior in the EPM and OFT compared to cocaine‐abstinent mice on a standard diet. While flavanols are often linked to anxiolytic effects (Jia et al. 2023; Stringer et al. 2015), certain methylxanthines—like caffeine—can induce anxiety‐like behavior depending on dose and baseline anxiety levels in preclinical models (Florén Lind et al. 2023; Gulick and Gould 2009). Importantly, the anxiogenic‐like effect in our study seems to reflect an interaction between cocoa intake and prior cocaine exposure, as cocoa consumption did not increase anxiety in cocaine‐naïve animals in our previous work (Melgar‐Locatelli, Mañas‐Padilla, Castro‐Zavala, et al. 2024). Therefore, cocoa may act not only as a reinforcer—as suggested by the increased consumption observed—but also as a modulator that exacerbates withdrawal‐related behavioral alterations such as anxiety and drug seeking.

Despite chronic cocaine exposure, no cognitive impairments were observed under our experimental conditions, contrasting with previous reports of cocaine‐induced persistent learning and memory deficits (Mañas‐Padilla et al. 2021). While high‐phenolic cocoa enhanced object memory in healthy animals (Melgar‐Locatelli, Mañas‐Padilla, Castro‐Zavala, et al. 2024), this effect was not fully confirmed here in cocaine‐abstinent mice, though a trend was observed. Additionally, although COC + COCOA mice were slower in the water maze, their traveled distance remained unchanged, suggesting no learning effects of cocoa.

Lastly, while some studies suggest females are more susceptible to cocaine's rewarding effects (Becker et al. 2012; Bobzean et al. 2010; Hilderbrand and Lasek 2014), the influence of sex on cognitive, emotional behavior, and AHN remains unclear (Tsao et al. 2023). In our study, no significant sex differences were found, though male mice exhibited lower locomotor activity during water maze habituation, suggesting subtle sex differences in activity levels without affecting cognitive performance.

In conclusion, our findings indicate that high‐polyphenol cocoa supplementation in cocaine‐abstinent mice enhances cocaine‐conditioned responses and intensifies subsequent anxiety‐like behavior. Although cocoa significantly promoted AHN, this enhancement was not sufficient to counteract the behavioral effects of cocaine. These results highlight the complex impact of natural cocoa on drug reward and associated anxiety. Our findings suggest, for the first time, a possible synergistic effect of cocoa and cocaine on the reward system, which opens a broad field of research into the relationships between diet and drug addiction, as well as the mechanisms involved in this synergy. Future studies should aim to more directly assess the reinforcing properties of natural cocoa to determine whether it can independently engage or modulate reward‐related neural circuits. Evaluating its impact on dopaminergic and glutamatergic signaling, as well as neuronal activation patterns (e.g., c‐Fos expression) in key regions of the reward circuitry such as the nucleus accumbens, ventral tegmental area, and prefrontal cortex would provide critical mechanistic insight. Such analyses may clarify whether cocoa acts merely as a modulatory factor or exerts a more direct influence on reward pathways.

## Author Contributions

**Sonia Melgar‐Locatelli:** conceptualization (equal), data curation (lead), formal analysis (lead), investigation (lead), methodology (lead), software (lead), validation (equal), visualization (equal), writing – original draft (lead), writing – review and editing (lead). **María del Carmen Mañas‐Padilla:** conceptualization (equal), methodology (equal), visualization (supporting). **Patricia Rivera:** investigation (equal), supervision (equal), validation (equal), visualization (equal), writing – review and editing (supporting). **Celia Rodríguez‐Pérez:** conceptualization (lead), funding acquisition (lead), investigation (equal), project administration (lead), resources (equal), supervision (equal), validation (equal), visualization (equal), writing – review and editing (equal). **Estela Castilla‐Ortega:** conceptualization (lead), funding acquisition (lead), investigation (equal), project administration (lead), supervision (lead), validation (equal), visualization (equal), writing – original draft (supporting), writing – review and editing (equal).

## Conflicts of Interest

The authors declare no conflicts of interest.

## Supporting information


**Data S1:** fsn370842‐sup‐0001‐Supinfo.pdf.

## Data Availability

Dataset available at Digibug: https://digibug.ugr.es/handle/10481/102997.
