# Supplementary material for: High‐Phenolic Cocoa Enhances Drug‐Induced Reinstatement of Cocaine‐Conditioned Place Preference Independently of Increasing Adult Hippocampal Neurogenesis
Source: Food Sci Nutr. 2025 Sep 14;13(9):e70842. doi: 10.1002/fsn3.70842 (PMC12434315; doi:10.1002/fsn3.70842)
Supplement: Supplementary file 1 — Data S1: fsn370842‐sup‐0001‐Supinfo.pdf. [file FSN3-13-e70842-s001.pdf]

Supplementary Material for:

High-phenolic cocoa enhances drug-induced reinstatement of cocaine-conditioned place preference independently of increasing adult hippocampal neurogenesis

Sonia Melgar-Locatelli*[1,2,3,4], María del Carmen Mañas-Padilla[1,2,5], Patricia Rivera[2,6,7], Celia Rodríguez-Pérez[3,4,8], Estela Castilla-Ortega*[1,2]

[1]Departamento de Psicobiología y Metodología de las Ciencias del Comportamiento, Universidad de Málaga, Málaga, Spain.

[2]Instituto de Investigación Biomédica de Málaga y Plataforma en Nanomedicina-IBIMA Plataforma BIONAND, Málaga, Spain.

[3]Departamento de Nutrición y Bromatología, Universidad de Granada, Campus Universitario de Cartuja, Granada, Spain.

[4]Instituto de Nutrición y Tecnología de los Alimentos 'José Mataix' (INYTA), Centro de Investigación Biomédica, Universidad de Granada, Granada, Spain.

[5]Universidad Internacional de la Rioja (UNIR), Rioja, Spain.

[6]Unidad de Gestión Clínica de Salud Mental, Hospital Regional Universitario de Málaga. Málaga, Spain.

[7]Departamento de Anatomía Humana, Medicina Legal e Historia de la Ciencia, Universidad de Málaga, Málaga, Spain.

[8]Instituto de Investigación Biosanitaria de Granada (ibs.GRANADA), 18012, Granada, Spain.

Correspondence to: Departamento de Psicobiología y Metodología de las CC, Facultad de Psicología, Universidad de Málaga, C. Dr. Ortiz Ramos, 29010 Málaga, Spain. Email address: soniaml@ugr.es (for S.M.L.); ecastilla@uma.es (for E.C.O.).

Supplementary Table 1

Table 1. Summary of statistical analyses that showed no significant effect for treatment or its interactions in cognitive and neurogenic assessments.

Test	Variable	Statistical Test	F Value	P Value
Elevated plus maze	Total locomotion	One-way ANOVA	F(2, 39) = 2.446	p = 0.100
Open field test	Total locomotion	One-way ANOVA	F(2, 39) = 0.064	p = 0.938
Recognition memory locomotion	Treatment	Repeated measures ANOVA	F(2,39) = 0.856	p = 0.433
	Session		F(2,78) = 2.163	p = 0.122
	Treatment x Session		F(4,78) = 0.235	p = 0.918
Recognition memory object exploration	Treatment	Repeated measures ANOVA	F(2, 39) = 2.405	p = 0.104
	Session		F(2, 78) = 7.286	p = 0.002
	Treatment x Session		F(4, 78) = 0.943	p = 0.444
Place memory recognition test	Object memory	One-way ANOVA	F(2, 39) = 1.193	p = 0.314
Object memory recognition test	Object memory	One-way ANOVA	F(2, 39) = 1.098	p = 0.344
Behavior during the forced swimming test	Treatment	Repeated measures ANOVA	F(2, 39) = 0.503	p = 0.608
	Behavior		F(2, 78) = 21.385	p < 0.001
	Treatment x Behavior		F(4, 78) = 0.235	p = 0.901
Habituation in the Water maze	Thigmotaxis	One-way ANOVA	F(2,39) = 2.051	p = 0.142
Locomotion during the visible platform in the Water maze	Treatment	Repeated measures ANOVA	F(2, 39) = 0.984	p = 0.383
	Trial		F(7, 273) = 21.581	p < 0.001
	Treatment x Trial		F(14, 273) = 1.790	p = 0.040
Locomotion during the reference memory training	Treatment	Repeated measures ANOVA	F(2, 39) = 2.542	p = 0.092
	Trial		F(23, 897) = 7.023	p < 0.001
	Treatment x Trial		F(46, 897) = 1.284	p = 0.101
Short-term memory retention	Treatment	Repeated measures ANOVA	F(2, 39) = 1.244	p = 0.299
	Quadrant		F(1,39) = 398.490	p < 0.001
	Treatment x Quadrant		F(2,39) = 0.313	p = 0.733
	Platform crossings	One-way ANOVA	F(2, 39) = 0.883	p = 0.422
Adult hippocampal neurogenesis markers	DCX	One-way ANOVA	F(2,39) = 0.618	p = 0.544
	PCNA	One-way ANOVA	F(2,39) = 0.885	p = 0.446

Supplementary Figure 1

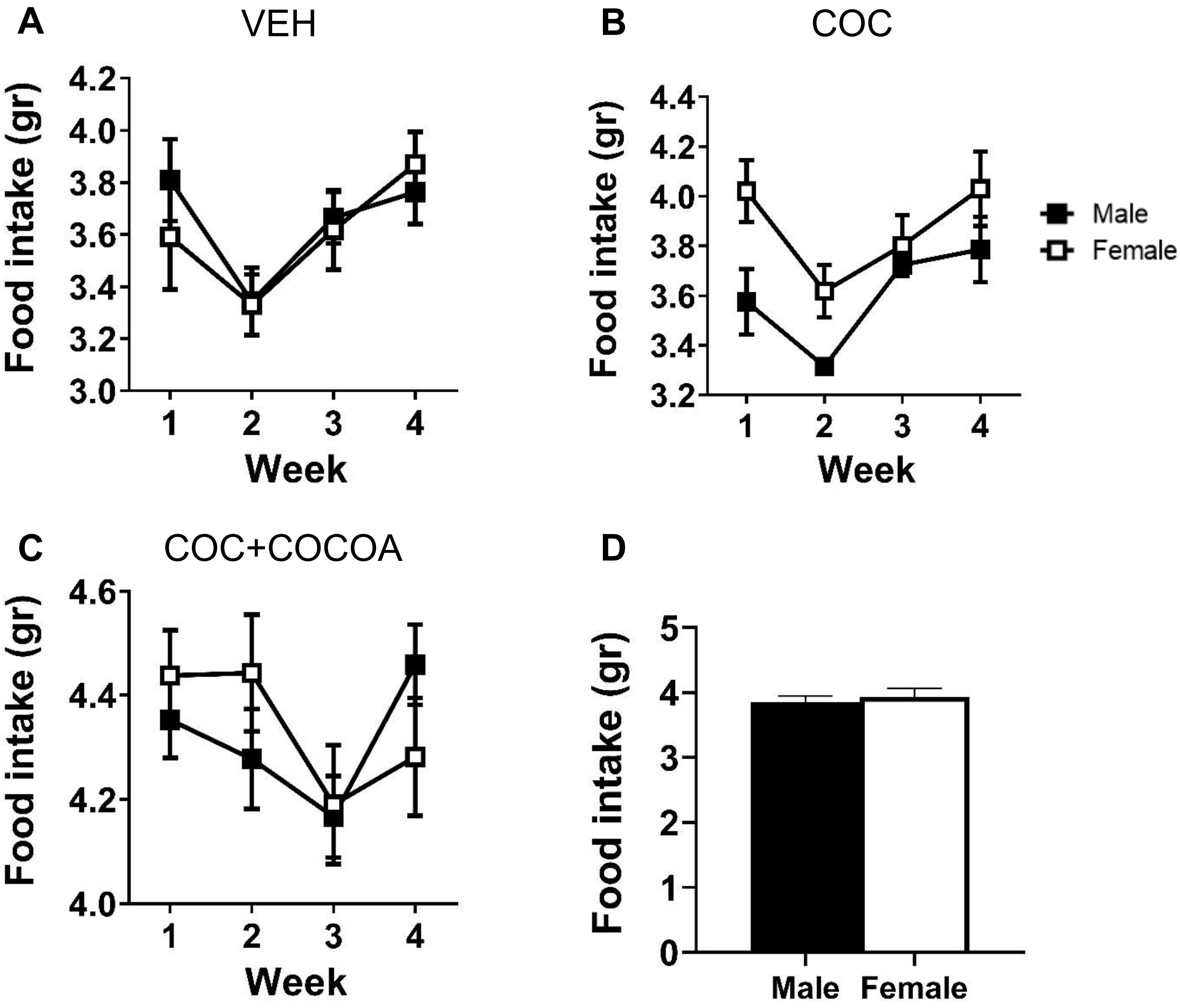

Supplementary Figure 1. Food intake differences with regard to sex. Food consumption was not influenced by the sex of the animals [repeated measures ANOVA 'treatment x week x sex' on food consumption: effect for 'treatment': $F(2, 36) = 34.403$, $p < 0.001$; 'session': $F(3, 108) = 10.410$, $p < 0.001$; 'sex': $F(1,36) = 1.240$, $p = 0.273$; 'treatment x session': $F(6, 108) = 3.085$, $p = 0.008$; 'treatment x sex': $F(2, 36) = 1.610$, $p = 0.214$; 'sex x session': $F(3, 108) = 0.478$, $p = 0.698$, 'treatment x sex x session': $F(6, 108) = 1.241$, $p = 0.291$]. For clarity, graphs A-C show data per individual diet treatment and week, while (D) shows data collapsed by sex. Results are expressed as mean ± SEM.

Supplementary Figure 2

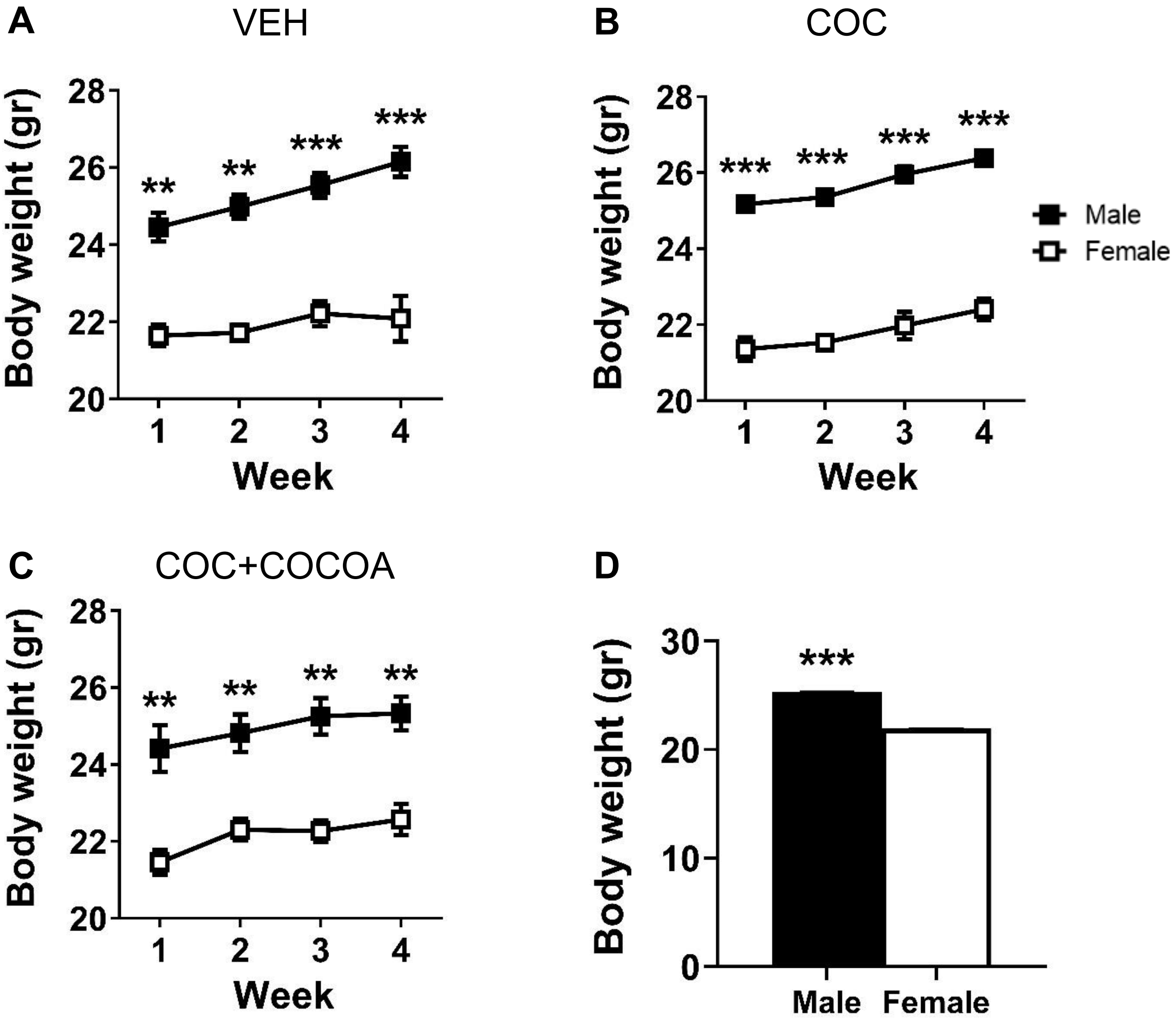

Supplementary Figure 2. Body weight differences with regard to sex. Irrespective of their dietary treatment, male mice exhibited a higher body weight compared to females [repeated measures ANOVA 'treatment x week x sex' on body weight data: effect for 'treatment': $F(2, 36) = 0.231$, $p = 0.795$; 'week': $F(3, 108) = 33.238$, $p < 0.001$; 'sex': $F(1,36) = 146.00$, $p < 0.001$; 'treatment x week': $F(6, 108) = 0.827$, $p = 0.552$; 'treatment x sex': $F(2, 36) = 1.302$, $p = 0.285$; 'sex x week': $F(3, 108) = 1.296$, $p = 0.280$, 'treatment x sex x week': $F(6, 108) = 1.193$, $p = 0.316$]. For clarity, graphs A-C show data per individual diet treatment and week, while (D) shows data collapsed by sex. Results are expressed as mean ± SEM; differences between sexes: **$p \leq 0.01$; ***$p \leq 0.001$.

Supplementary Figure 3

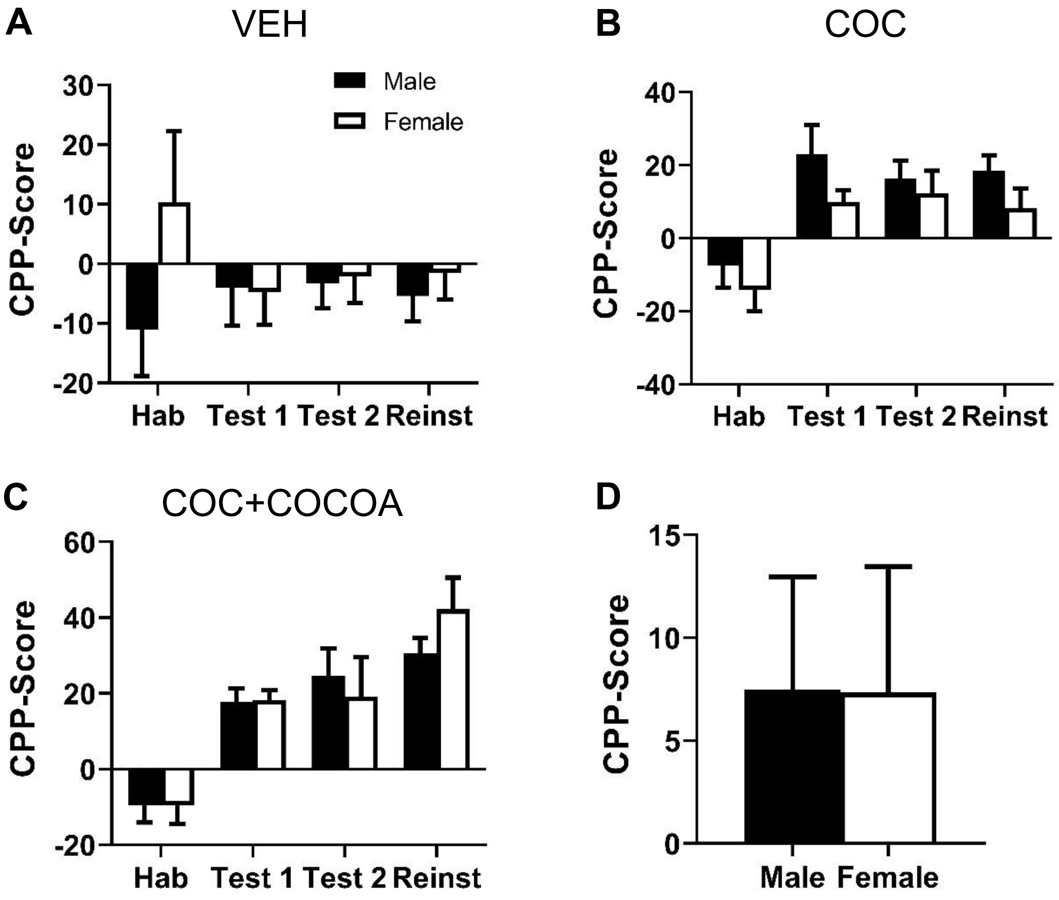

Supplementary Figure 3. No sex differences were found in the CPP-Score [repeated measures ANOVA ‘treatment x session x sex’ on CPP-Score: effect for ‘treatment’: $F(2, 36) = 11.088$, $p < 0.001$; ‘session’: $F(3, 108) = 20.589$, $p < 0.001$; ‘sex’: $F(1,36) = 0.002$, $p = 0.964$; ‘treatment x session’: $F(6, 108) = 8.741$, $p < 0.001$; ‘treatment x sex’: $F(2, 36) = 1.700$, $p = 0.197$; ‘sex x session’: $F(3, 108) = 0.940$, $p = 0.424$, ‘treatment x sex x session’: $F(6, 108) = 1.033$, $p = 0.408$]. For clarity, graphs A-C show data per individual diet treatment and session, while (D) shows data collapsed by sex. Results are expressed as mean ± SEM.

Supplementary Figure 4

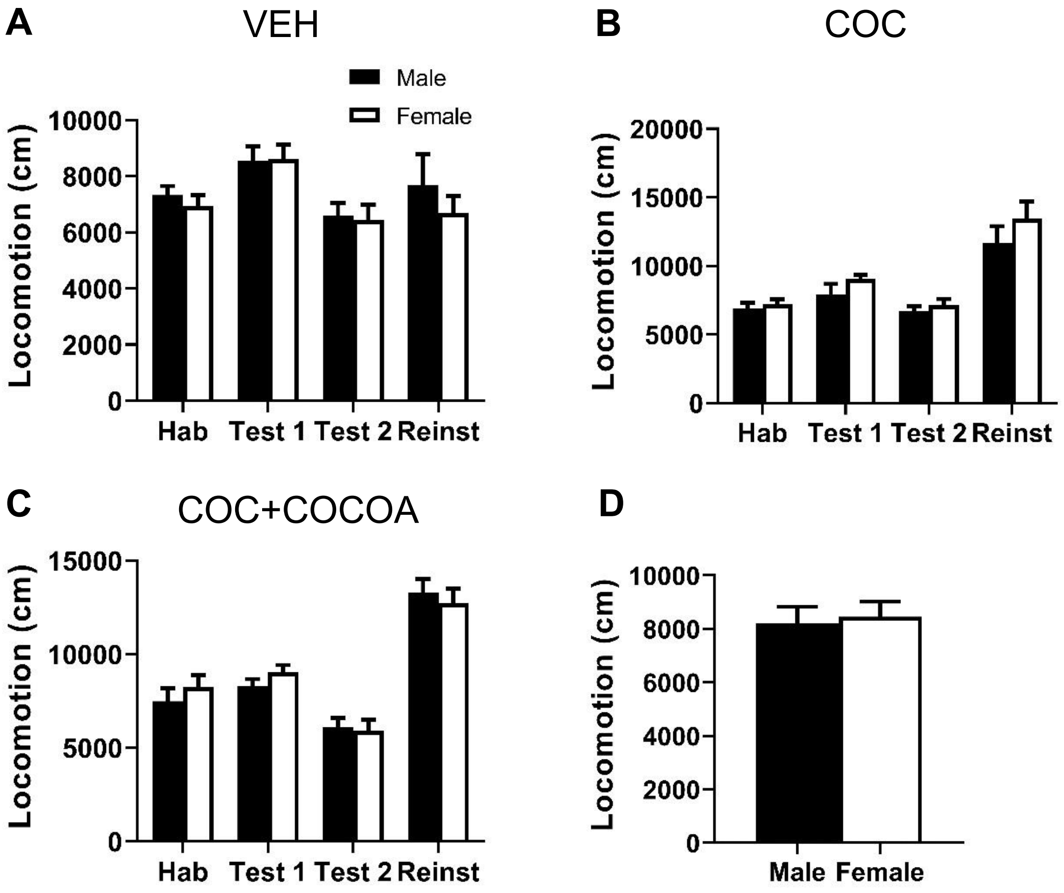

Supplementary Figure 4. No sex differences were found in locomotion across the CPP task [repeated measures ANOVA 'treatment x session x sex' on locomotion: effect for 'treatment': $F(2, 36) = 9.051$, $p < 0.001$; 'session': $F(3, 108) = 63.082$, $p < 0.001$; 'sex': $F(1,36) = 0.587$, $p = 0.449$; 'treatment x session': $F(6, 108) = 15.207$, $p < 0.001$; 'treatment x sex': $F(2, 36) = 1.319$, $p = 0.280$; 'sex x session': $F(3, 108) = 0.355$, $p = 0.785$, 'treatment x sex x session': $F(6, 108) = 0.596$, $p = 0.733$]. For clarity, graphs A-C show data per individual diet treatment and session, while (D) shows data collapsed by sex. Results are expressed as mean ± SEM.

Supplementary Figure 5

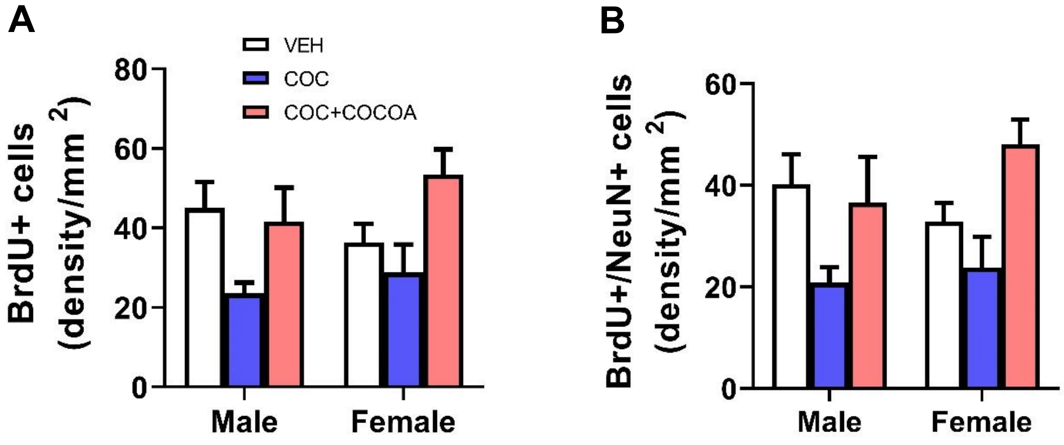

Supplementary Figure 5. Male and female mice showed comparable numbers of BrdU+ cells (A) [one-way ANOVA: $F(1, 37) = 0.045$, $p = 0.947$], as well as similar differentiation into mature neurons (BrdU+/NeuN+; B) [one-way ANOVA: $F(1, 37) = 0.072$, $p = 0.790$]. Data are presented as mean ± SEM.

Supplementary Figure 6

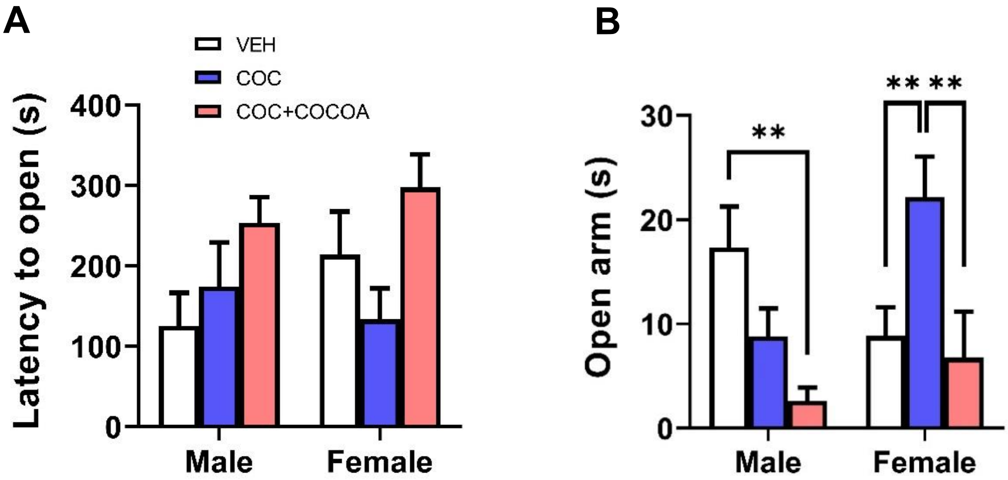

Supplementary Figure 6. No overall sex differences were observed in anxiety-like behavior in the EPM, as indicated by similar latencies to enter the open arms (A) [one-way ANOVA: $F(1, 40) = 0.611$, $p = 0.439$]. However, control females spent less time in the open arms than males, but this pattern was reversed with cocaine: females showed increased open-arm time, while males showed a reduction. In the cocoa-treated group, both sexes displayed similarly low open-arm times. Results are expressed as mean ± SEM. Group differences across sexes:**$p \leq 0.01$

Supplementary Figure 7

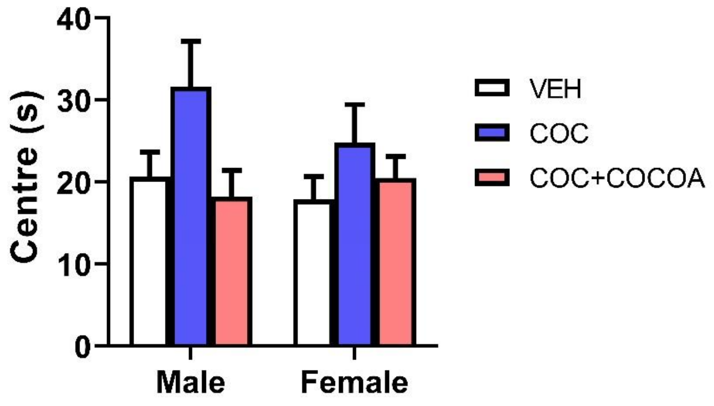

Supplementary Figure 7. During OFT habituation, all female and male mice spent a similar amount of time in the center of the apparatus [one-way ANOVA: $F(1, 40) = 0.565$, $p = 0.456$]. Results are expressed as mean ± SEM.

Supplementary Figure 8

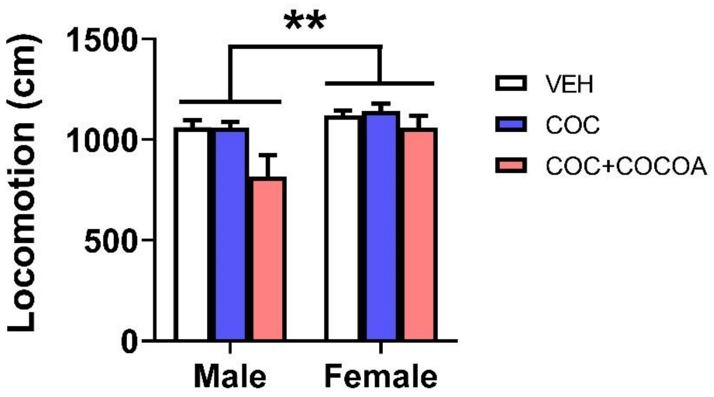

Supplementary Figure 8. During water maze habituation, male mice exhibited significantly reduced locomotor activity compared to female mice. Data are presented as mean ± SEM. Differences between sexes: $^{**}p \leq 0.01$.
